# Retrieval of Suspended Sediment Concentration from Bathymetric Bias of Airborne LiDAR

**DOI:** 10.3390/s222410005

**Published:** 2022-12-19

**Authors:** Xinglei Zhao, Jianfei Gao, Hui Xia, Fengnian Zhou

**Affiliations:** 1 College of Information Science and Engineering, Shandong Agricultural University, Tai’an 271018, China; 2 The Survey Bureau of Hydrology and Water Resources of Yangtze Estuary, Shanghai 200136, China

**Keywords:** airborne LiDAR bathymetry, green laser, suspended sediment concentration, depth bias, near-water-surface penetration

## Abstract

In addition to depth measurements, airborne LiDAR bathymetry (ALB) has shown usefulness in suspended sediment concentration (SSC) inversion. However, SSC retrieval using ALB based on waveform decomposition or near-water-surface penetration by green lasers requires access to full-waveform data or infrared laser data, which are not always available for users. Thus, in this study we propose a new SSC inversion method based on the depth bias of ALB. Artificial neural networks were used to build an empirical inversion model by connecting the depth bias and SSC. The proposed method was verified using an ALB dataset collected through Optech coastal zone mapping and imaging LiDAR systems. The results showed that the mean square error of the predicted SSC based on the empirical model of ALB depth bias was less than 2.564 mg/L in the experimental area. The proposed method was compared with the waveform decomposition and regression methods. The advantages and limits of the proposed method were analyzed and summarized. The proposed method can effectively retrieve SSC and only requires ALB-derived and sonar-derived water bottom points, eliminating the dependence on the use of green full-waveforms and infrared lasers. This study provides an alternative means of conducting SSC inversion using ALB.

## 1. Introduction

The suspended sediment concentration (SSC), the mass of the sediment entrained within a unit of water volume (mg/L), is a common measure of sediment transport. Acquiring reliable and spatially distributed observations of SSC, which plays a major role in erosion/deposition processes; biomass primary production; and the transport of nutrients, micropollutants, and heavy metals, is important in order to advance our understanding of the biogeomorphic dynamics of estuarine and lagoon systems [1]. However, the acquisition of SSC values with high accuracy, high temporal and spatial resolution, a large area, and cost effectiveness is not an easy goal to achieve. At present, SSC acquisition or water quality monitoring methods mainly include the in situ sampling method, the satellite remote sensing method [2,3,4,5,6,7,8], and the airborne LiDAR bathymetry (ALB) inversion method [9,10,11,12]. In the in situ sampling method, a water sample is most commonly collected in the field and filtered to extract suspended matter. The filtered material is then dried, weighed, and divided by the sample volume to obtain the SSC. The in situ sampling method has the advantage of high accuracy, but its temporal and spatial resolution and efficiency are low. Satellite remote sensing can achieve large-area SSC inversion, but its temporal and spatial resolution and accuracy are low [2].

ALB technology uses an airborne laser sensor to emit green laser pulses (λ = 532 nm) and receive pulse returns to detect the water surface, water body, and water bottom. The primary goal of ALB system design is high-accuracy and high-resolution depth measurements for shallow waters [13]. Some ALB systems emit an additional infrared laser to detect the water surface and overcome the water surface uncertainty problem which occurs with the use of green lasers [13,14]. ALB systems using only green lasers and integrated infrared and green lasers are called single-wavelength and dual-wavelength ALB systems, respectively. In addition to basic depth measurements, ALB technology has been applied to SSC retrieval [9,10,11]. SSC has a significant impact on the ALB waveform shape and measurement accuracy. Conversely, SSC inversion can be achieved by analyzing ALB waveform shape features or measurement bias.

At present, there are two main methods used to retrieve SSC using ALB information: the waveform decomposition method and the measurement bias method. The waveform decomposition method [10] uses waveform features of volume backscatter return (VBR) which are related to SSC to retrieve the SSC. The measurement bias method [11] uses point cloud bias information to retrieve the SSC. Laser waveform data are the raw ALB measurement data which reflect return pulse intensity, and the three-dimensional point cloud data are the products obtained by merging the waveform and corresponding position and orientation data [15].

The typical green laser bathymetric waveform consists of three parts: the air-water interface return (AIR), the VBR, and the bottom return (BR). The surface return is a linear superposition of the AIR and VBR [13]. The VBR is significantly affected by SSC; thus, its waveform parameters can be used to retrieve SSC [10]. Waveform decomposition should be conducted first to extract the VBR from the superposed bathymetric waveform [8]. At present, waveform decomposition methods for the green waveforms of ALB can be classified into two types: Gaussian decomposition [16,17,18,19] and AIR, VBR, and BR (AVB) decomposition [20,21,22,23]. Gaussian decomposition is oriented towards topographic LiDAR waveforms and transplanted to bathymetric waveforms [16,17]. The sum of several Gaussian functions can ensure good fitness based on the fitting principle but cannot well represent VBR, which is a continuous return that is backscattered from suspended sediments [23]. AVB decomposition methods have been proposed based on fitting the three returns using three different functions. AVB decomposition methods have shown their ability to process green waveforms and extract VBRs from superposed green waveforms. The amplitude and slope of the VBR, extracted using waveform decomposition, have been used to retrieve SSC values [10]. The waveform decomposition method has been verified as an effective method for SSC retrieval using VBR, but full-waveform data are not always available for users; thus, this method is limited in practical applications.

Because of the water surface uncertainty problem, green lasers can hardly detect the water surface accurately but can penetrate to a certain depth under the water surface [24]. This height bias of the green surface point is called near-water-surface penetration (NWSP) [25]. The NWSP of a green laser is significantly affected by the SSC of the surface layer. Conversely, if the NWSP of the green laser is known, the SSC of the surface layer can be estimated using NWSP as an indicator. Based on this idea, Zhao et al. proposed a method for SSC inversion of the water surface layer using the NWSP of green lasers for dual-wavelength ALB systems [11]. This method uses the water surface point, accurately detected by an infrared laser, as a reference to calculate the NWSP of a green laser and effectively realizes SSC inversion by constructing an empirical SSC model of the NWSP. However, this method requires the help of an infrared laser and is only applicable to dual-wavelength ALB systems. Similar to the height bias of the water surface point (NWSP) derived by the green laser, the height bias of the water bottom points (depth bias) derived by the green laser is also significantly affected by SSC [26]. If the ALB depth bias can be calculated, SSC can be retrieved by establishing an empirical model of the depth bias. Moreover, the calculation of the height bias of the water bottom point does not require the help of an infrared laser, and it can be used to realize SSC inversion in a manner which is generally suitable for single- and dual-wavelength ALB systems. Therefore, this paper presents a general SSC inversion method, i.e., SSC inversion based on the depth bias of ALB, that can be applied to single- and dual-wavelength ALB systems without the aid of infrared lasers, and which provides theoretical and methodological support for solving the problem of SSC observation in coastal shallow waters.

## 2. Methods

**Figure 1:**
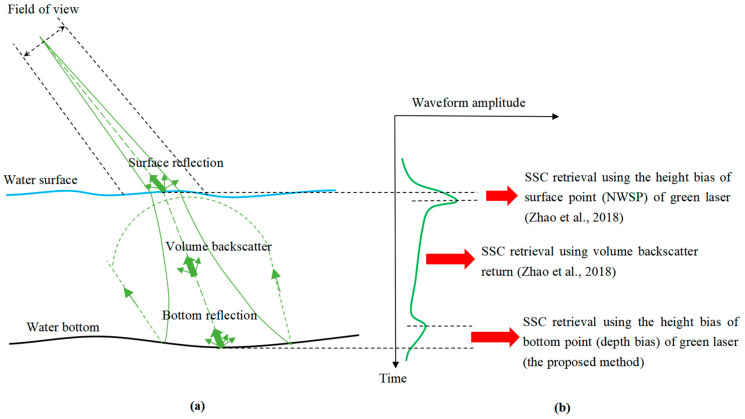
Illustration of SSC retrieval using ALB: (**a**) green laser propagation, (**b**) SSC retrieval using ALB [10,11].

Figure 1a shows an illustration of airborne green laser propagation, and Figure 1b shows the corresponding laser waveform, representing the return waveform amplitude of the laser’s interaction with the water surface, the water body, and the water bottom. SSC retrieval using ALB focuses on the laser’s measurement bias and the laser waveform, which represent the interaction of laser pulses and ocean environments.

### 2.1. Waveform Decomposition Method

Zhao et al. deduced that water turbidity is related to the amplitude and slope of the VBR of the laser’s bathymetric waveform by means of the bathymetric LiDAR equation [10]. Thus, the amplitude or slope of the VBR can be used to indicate water turbidity or SSC. However, the raw bathymetric laser waveform is the superposition of the AIR, VBR, and BR. Waveform decomposition should be conducted to extract the VBR from the superposed waveform. Traditional decomposition methods do not handle bound constraints and are easily trapped in local optima; thus, the decomposed components may be inconsistent with the measurement principles of ALB. Zhao et al. proposed a constrained waveform decomposition method by setting reasonable lower and upper bounds on waveform parameters to guarantee the fidelity of the decomposed components [23].

AIR is repressed as a Gaussian function:

(1) $$\mathrm{AIR}\left(t;A_{s},t_{s},\sigma_{s}\right)=A_{s}\exp\left[\frac{-{\left(t-t_{s}\right)}^{2}}{2\sigma_{s}^{2}}\right]$$

where *t* is the time and *A_s_*, *t_s_*, and *σ_s_* are the amplitude, peak position, and standard deviation of the AIR, respectively. Typically, the VBR is expressed as a triangular function:

(2) $$\mathrm{VBR}\left(t;A_{c},a,b,c\right)=A_{c}\times \left\{\begin{array}{cc} 0 & t\leq a\\ \frac{t-a}{b-a} & a\leq t\leq b\\ \frac{c-t}{c-b} & b\leq t\leq c\\ 0 & c\leq t \end{array}\right.$$

where *A*_c_, *a*, *b*, and *c* are the amplitude, start, peak, and end position of the VBR, respectively. BR is expressed as a Gaussian function if it exists in the raw waveform.

(3) $$\mathrm{BR}\left(t;A_{b},t_{b},\sigma_{b}\right)=A_{b}\exp\left[\frac{-{\left(t-t_{b}\right)}^{2}}{2\sigma_{b}^{2}}\right]$$

where *A_b_*, *t_b_*, and *σ_b_* are the amplitude, peak position, and standard deviation of the BR, respectively. The constrained nonlinear optimization can be transformed into the following form:

(4) $$\hat{\beta }=\arg\min_{\beta }\left\{\sum_{i=1}^{m}{\left(y_{i}-f\left(t_{i};\beta \right)\right)}^{2}\;\mathrm{such}\;\mathrm{that}\;l\leq \beta \leq u\right\}$$

where *l* and *u* are vectors of the lower and upper bounds of the waveform parameters, respectively. The amplitude *A*_C_ and slope *K* = *A*_C_/(*c* − *b*) of the VBR can be obtained using constrained waveform decomposition and these can be used as indicators to retrieve the SSC. The waveform decomposition method has been verified as an effective method for SSC retrieval using airborne bathymetric LiDAR data [10]. However, full-waveform data are not always available for users; thus, this method is limited in its practical applications.

### 2.2. Measurement Bias Method

The measurement biases of the green laser reflect the features of the green laser’s waveform, i.e., time bias features. The height bias of the surface point (NWSP) of the green laser reflects the time bias of the surface peak, and the height bias of the bottom point (depth bias) of the green laser reflects the time bias of the bottom peak. The time bias of the surface peak is mainly reduced due to the water surface uncertainty problem of the green laser [24]. The time bias of the bottom peak is mainly reduced due to the pulse stretching effect [27], which causes the peak position of the bottom peak to deviate from the correct bottom position. The calculation of the NWSP requires the use of an infrared laser and is not applicable for single-wavelength ALB systems.

Taking the height of the water bottom point *h*_0_ obtained by single-beam or multibeam echo sounders as a reference, the depth bias Δ*h* of the water bottom point derived by the green laser can be expressed as [26]:

(5) $$\Delta h=h_{ALB}-h_{0}$$

where *h*_ALB_ represents the height of the water bottom point derived by the green laser. Previous studies have shown that the depth bias of ALB varies with water depth [28,29]. The rate of change in Δ*h* with water depth is mainly affected by the SSC, beam scanning angle, and sensor height. Zhao et al. used the stepwise regression method to build a depth bias model considering SSC, which improved the ALB sounding accuracy [26] as follows:

(6) $$\begin{array}{c} \Delta h=\mu D+b\\ \mu =\beta_{1}+\beta_{2}\theta +\beta_{3}\theta^{2}+\beta_{4}H^{2}+\beta_{5}C \end{array}$$

where *b* is a constant term, *β*_1_–*β*_5_ are the model coefficients, *C* is the SSC, *θ* is the beam scanning angle, and *H* is the sensor height. Conversely, the SSC can be estimated if Δ*h* is known. Compared with SSC inversion using the NWSP derived by means of a green laser, this method does not require the water surface point to be derived using an infrared laser and can realize SSC inversion in a manner that is suitable for single- and dual-wavelength ALB systems.

The ALB laser spot and the single-beam sounding point with approximately the same position are called a point pair. The Δ*h* of each point pair can be calculated using Equation (5). *D* can be calculated by subtracting the height of the water bottom point, derived via sonar measurements, from the height of the water surface point, derived via ALB. The beam scanning angle *θ* and the sensor height *H* can be extracted from the raw ALB data. Affected by the complex environment and measurement parameters, the accurate response mechanism of the depth bias to the SSC is difficult to express. Therefore, the relationship between the measured SSC at each sampling station and the corresponding depth bias of the green laser needs to be deeply explored, and an empirical SSC model of the ALB depth bias needs to be constructed. Artificial neural networks (ANNs) were used to build the SSC model in this study. The structure of the ANN-based SSC model of ALB depth bias includes the input layer, hidden layer, and output layer. Among these, the input layer includes the factors influencing SSC, including the depth bias of ALB, water depth, beam scanning angle, and sensor height. The hidden layer consists of multiple neurons, and the output layer is the predicted SSC. After obtaining the reliable empirical SSC model, the SSC of each laser spot can be calculated using the constructed SSC model and the ALB depth bias. The mean square error (MSE) and correlation coefficient *R* are used to evaluate the performance of the SSC empirical model.

(7) $$\mathrm{MSE}=\frac{1}{m}\sum_{i=1}^{m}{\left(f\left(x_{i}\right)-y_{i}\right)}^{2}$$

(8) $$R=\frac{\sum_{i=1}^{m}\left(f(x_{i})-\bar{f}\right)\left(y_{i}-\bar{y}\right)}{\sqrt{\sum_{i=1}^{m}{\left(f(x_{i})-\bar{f}\right)}^{2}}\sqrt{\sum_{i=1}^{m}{\left(y_{i}-\bar{y}\right)}^{2}}}$$

where *m* is the total number of point pairs, *i* is the *i*th point pair ranging from 1 to *m*, *f* is the SSC predicted by the ANN-based model, and *y* is the measured SSC.

## 3. Experiment and Results

### 3.1. Research Area and Data Acquisition

**Figure 2:**
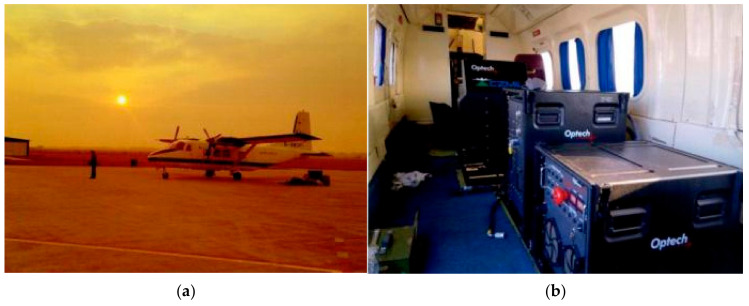
ALB system used in the experiment: (**a**) Y-12 aircraft, (**b**) Optech CZMIL.

**Figure 3:**
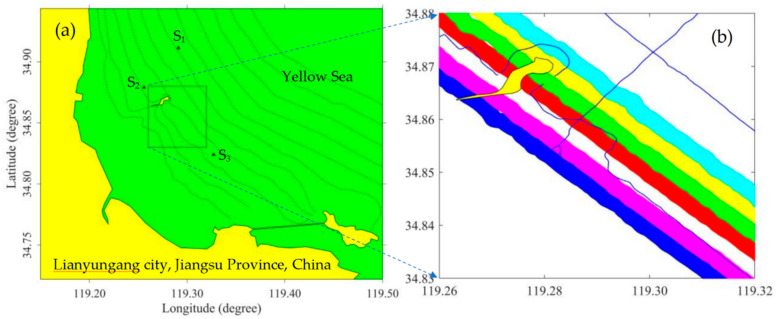
Locations of the ALB and sonar measurements. (**a**) Locations and (**b**) local enlarged view of the research area.

An ALB measurement was carried out using the Optech coastal zone mapping and imaging LiDAR (CZMIL) system in the coastal waters of Lianyungang city, Jiangsu Province, China, to verify the effectiveness of the SSC retrieval method. Figure 2 shows the manned Y-12 aircraft and Optech CZMIL used in the experiment. CZMIL is a dual-wavelength ALB system that adopts collinear and synchronous means to emit IR (1064 nm) and green (532 nm) lasers [30,31]. Sounding measurements were carried out using an HY-1600 single-beam sounding sonar simultaneously with the ALB measurements to provide reference bottom points for ALB depth bias calculations. The locations of the ALB and sonar measurements are shown in Figure 3. The yellow and green colors in Figure 3a represent the land and the Yellow Sea, respectively. The black triangles represent the locations of the three SSC sampling stations. The SSCs of the S_1_, S_2_, and S_3_ sampling stations were 315, 122, and 134 mg/L, respectively. Figure 3b presents an enlarged drawing of the red boxed area in Figure 3a. The blue, magenta, red, green, yellow, and cyan colors represent six strips of ALB measurements. The black curve represents Qinshan Island. The blue dotted lines represent the tracklines of single-beam echo sounding. Detailed descriptions of the experimental area and the Optech CZMIL system can be found in [26].

### 3.2. Data Preparation

**Figure 4:**
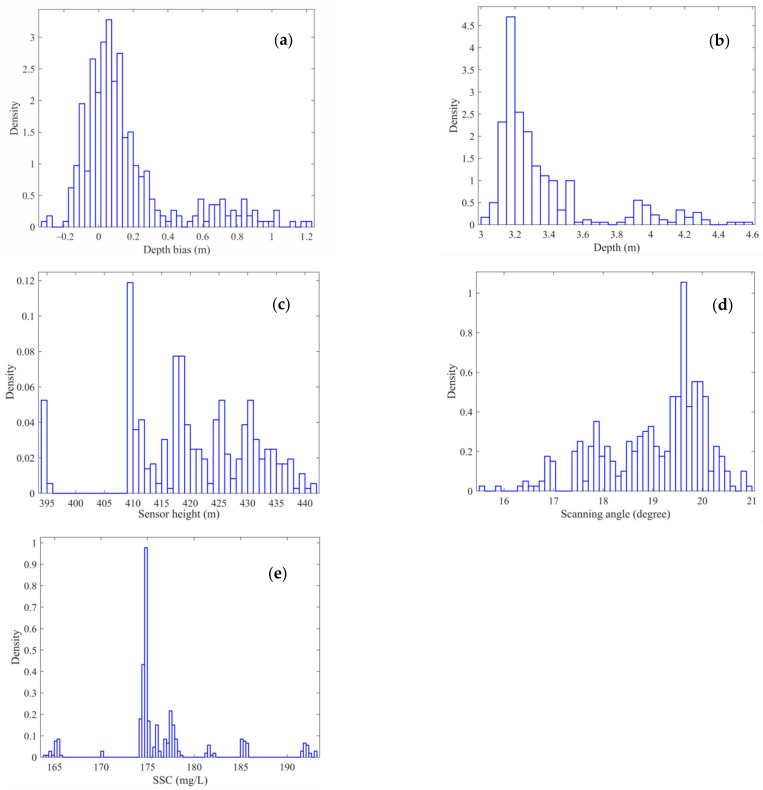
Probability density distribution of the model input and output data. (**a**) Depth bias, (**b**) depth, (**c**) sensor height, (**d**) beam scanning angle, and (**e**) SSC.

**Figure 5:**
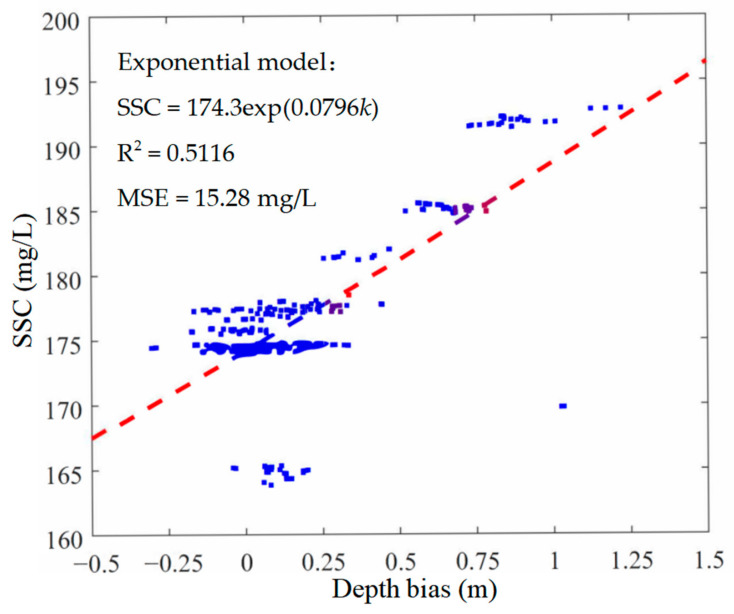
Relationship of ALB depth bias and SSC and the fitted exponential model.

First, data preprocessing was performed on the ALB raw data and the single-beam sounding data. Then, the ALB and the single-beam point pair at the common position were searched according to the position information. In the experimental area, there were 362 pairs of ALB and sonar sounding points with approximate common positions. According to Equation (5), the ALB depth bias can be calculated at a common position with the elevation of the single-beam bottom point as the reference. The SSC of each point pair can be estimated using the measured SSC from the sample stations based on the inverse distance weight algorithm. The 362 point pairs were randomly divided into training (60%), validation (20%), and testing (20%) data. The training data were presented to the network during training, and the network was adjusted according to its error. The validation data were used to measure network generalization and to halt training when the generalization process stopped improving. The testing data had no effect on training and thus provided an independent measure of network performance during and after training. The probability density distributions of the raw depth bias of ALB, water depth, sensor height of the ALB system, laser beam scanning angle, and SSC of the point pairs are shown in Figure 4a–e, respectively. The statistical parameters of these raw data are listed in Table 1. As shown in Figure 5, the relationship between the raw ALB depth bias and measured SSC presented an approximate positive correlation, i.e., the higher the SSC, the larger the ALB depth bias, indicating that the ALB depth bias was greatly influenced by the SSC. Furthermore, the distribution shown in Figure 5 presented divergent characteristics because ALB depth bias was influenced not only by SSC but also by other environmental and measurement parameters, such as the water depth, sensor height, and beam scanning angle.

### 3.3. SSC Modeling and Verification

**Figure 6:**
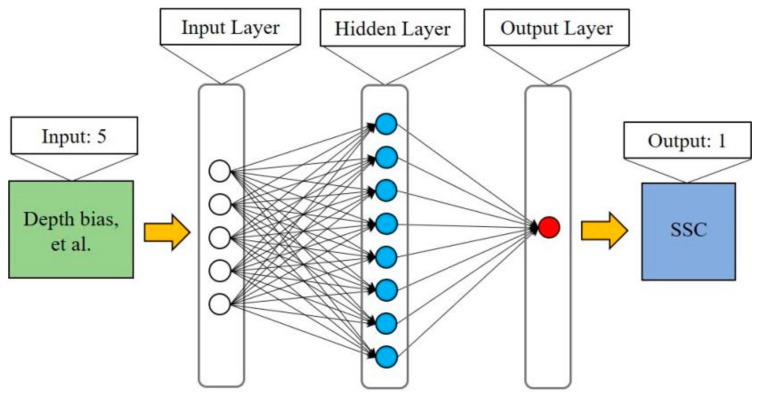
Structure of the ANN-based SSC model.

As shown in Figure 6, the structure of the constructed ANN model consists of three layers of neurons: an input layer, a hidden layer, and an output layer. The input layer contains five parameters, namely, Δ*h*, *D*cos*θ*, *D*cos^2^*θ*, *DH*, and *DH*^2^. The hidden layer contains 20 neurons, and the output layer outputs the predicted value of the SSC. Neurons are connected in a feed-forward fashion with input neurons that are fully connected to neurons in the hidden layer and hidden neurons that are fully connected to neurons in the output layer [32]. The activation function, also called the transfer function, is used to transform the activation level of neuron *x* into an output signal [33]. The objective of the nonlinear activation function was to introduce non-linearity into the network. Without non-linearity, a neural net is unable to handle complex modeling problems [32]. The sigmoid symmetric function *tansig* is a commonly used activation function, and this was applied in our network as follows:

(9) $$tansig\left(x\right)=\frac{2}{1+e^{-2x}}-1$$

The Bayesian regularization algorithm was used to train the network because it has been found to result in good generalizations for difficult, small, or noisy datasets [34].

**Figure A1:**
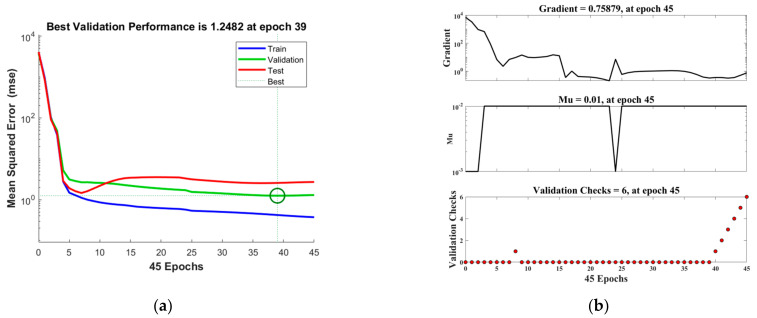
Training state of the SSC model: (**a**) MSE, (**b**) gradient, Mu, and validation checks.

**Figure A2:**
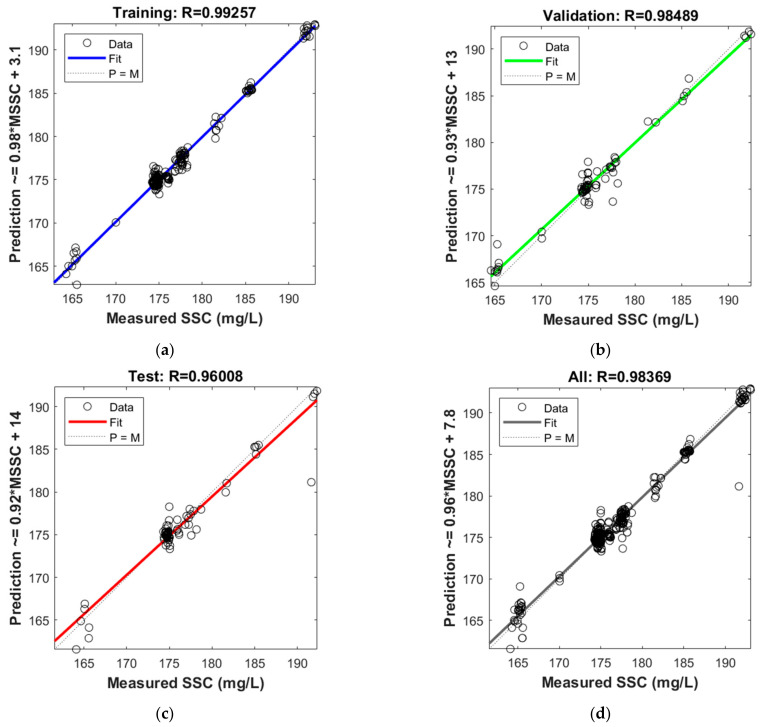
Regression of SSC prediction results: (**a**) regression of training data, (**b**) regression of validation data, (**c**) regression of test data, (**d**) regression of all data.

If the mean square error (MSE) increased six consecutive times (validation checks = 6), the number of iterations reached 1000, or the MSE reached zero, the network stopped training. In our experiment, the training stopped at 45 iterations because there were six validation checks. The training state of the ANN-based SSC model and the regression of the model results are shown in the Appendix A. The best validation performance (mean squared error) was 1.2482 at epoch 39. The gradient, Mu, and validation checks varying with epoch are shown in Figure A1b of Appendix A. The regression of the measured SSC and the SSC predicted based on the training data, the validation data, the testing data, and all data are shown in Figure A2a–d of Appendix A, respectively. As shown in Table 2, the MSEs of the training, validation, and testing data were 0.421, 1.248, and 2.564 mg/L, respectively; the *R* values of the training, validation, and testing data were 0.993, 0.985, and 0.960, respectively. The low MSE and high *R* values indicate that the ANN-based SSC model constructed using ALB depth bias as its input had high SSC inversion accuracy. Accuracy evaluations using MSE and *R* enabled us to ensure the reliability of the SSC empirical model.

### 3.4. SSC Inversion

**Figure 7:**
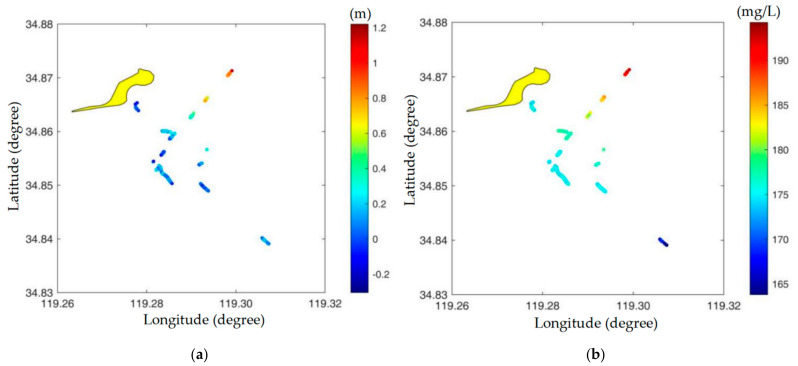
Spatial distributions of depth bias and the retrieved SSC. (**a**) Depth bias of ALB, and (**b**) retrieved SSC.

When the ANN-based SSC empirical model was obtained, the SSC of the research area could be estimated by inputting the input parameters into the constructed SSC model. The spatial distributions of the depth bias of the point pairs from the research area are shown in Figure 7a. The colors of discrete points represent depth bias values. In the research area, the spatial distributions of the SSC retrieved by inputting ALB depth bias into the constructed SSC model are shown in Figure 7b. The colors of the discrete points represent the retrieved SSC values.

## 4. Discussion

### 4.1. Comparison Methods

#### 4.1.1. Exponential SSC Regression Model of Depth Bias

As discussed in Section 3.2, the relationship between raw ALB depth bias and SSC presented an approximate positive correlation. This relationship can be modeled by fitting an exponential function *ψ* to the raw data. The parameters *η* of the exponential function can be estimated based on the least squares method as follows:

(10) $$\hat{\eta }=\arg\min_{\eta }\left\{\sum_{k=1}^{n}{\left(\psi_{k}\left(\eta \right)-SSC_{k}\right)}^{2}\;\right\}$$

where *k* represents ALB depth bias values and *n* is the total number of depth biases. The red dotted curve shown in Figure 5 represents the fitted exponential SSC model as follows:

(11) $$\mathrm{SSC}=174.3\exp\left(0.0796k\right)$$

The MSE and R^2^ of the fitted exponential SSC model of ALB depth bias were 15.28 mg/L and 0.5116, respectively.

#### 4.1.2. Waveform Decomposition Method

**Figure 8:**
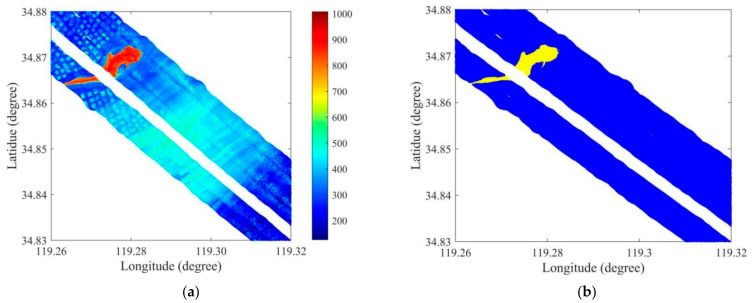
Spatial distributions of ocean and land waveforms. (**a**) Amplitudes of IR waveforms, and (**b**) laser spot positions of corresponding separated ocean (blue) and land (yellow) waveforms.

**Figure 9:**
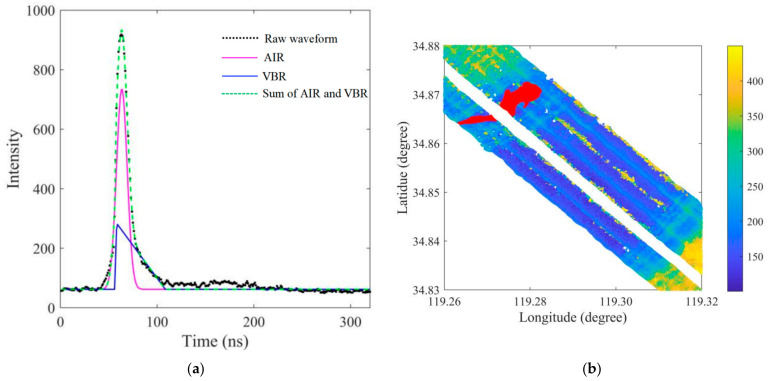
Waveform decomposition of a typical waveform and the distribution of VBR amplitudes. (**a**) Waveform decomposition, and (**b**) VBR amplitude.

**Figure 10:**
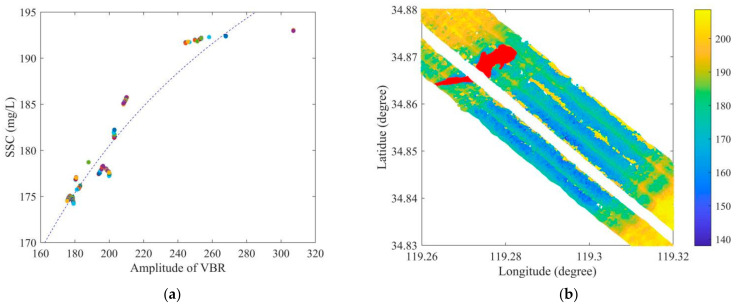
Distribution of VBR amplitude and retrieved SSC. (**a**) Relationship between SSC and VBR amplitude, and (**b**) retrieved SSC using the empirical model.

The waveform parameters of the VBR in ALB waveforms were theoretically analyzed and verified as effective indicators of SSC. An empirical model was built by connecting the waveform parameters of the VBR and the measured SSC to invert the SSC [10]. The procedure involved in the waveform decomposition method can be summarized as follows:

(1) Waveform extraction
  The raw laser waveforms collected by ALB systems are usually stored in binary files to save storage space. The raw data files must be decoded according to the data file format to extract all useful parameters and raw waveform data.

(2) Ocean-land waveform classification
  ALB systems can realize integrated ocean and land measurements based on the received laser pulse returns reflected from the ocean and land. Ocean-land waveform classification should be conducted to identify the ocean waveforms from the raw collected waveforms. Ocean-land waveform classification methods have been summarized and the dual-clustering method has been proposed as an effective method, with high accuracy for dual-wavelength ALB systems [35]. The dual-clustering method was used for ocean-land waveform classification in this study. The amplitudes of the IR waveforms were calculated and these are shown in Figure 8a. The yellow and blue colors in Figure 8b represent the spatial distributions of the obtained land and ocean waveforms, respectively.

(3) Ocean waveform decomposition
  Waveform decomposition, which is achieved by fitting the mathematical waveform model to raw green waveforms using a nonlinear fitting approach, is a powerful tool to extract the VBR from raw bathymetric waveforms. An improved AVB decomposition method—setting reasonable lower and upper bounds of waveform parameters—has been proposed to guarantee the fidelity of the decomposed components [23]. The AVB decomposition method was performed on the ocean waveforms classified in step 2 to extract the VBR of each pulse waveform. Figure 9a shows the waveform decomposition results for a typical bathymetric waveform in the research area. The black discrete points represent the pulse return intensity with a sampling period of 1 ns. The magenta and blue curves represent the AIR and VBR, respectively. The green dotted line represents the sum of the AIR and VBR. The bottom return is missing in the raw waveform because of the high turbidity. The distribution of the amplitudes of the extracted VBRs for the entire research area is shown in Figure 9b. Although the AVB decomposition method has shown its effectiveness for VBR extraction [10], the decomposition accuracy of some waveforms was low and should be improved further, e.g., the amplitudes of VBRs at the edge of some strips shown in Figure 9b were significantly larger than those of the adjacent areas.

(4) Empirical model construction and SSC retrieval
  The SSCs at the positions of the previously described point pairs and corresponding amplitudes of VBRs are shown in Figure 10a. The results showed that the SSCs and VBR amplitudes presented a positive correlation. A power function was used to build an SSC model of the VBR amplitude, similarly to Equation (10), based on the least-squares method. As indicated by the blue dotted line in Figure 10a, the fitted-power empirical SSC model of the VBR amplitude is expressed as follows:
  (12) $$\mathrm{SSC}=-3564\mu^{–0.7687}+241.2$$
  where *μ* is the amplitude of VBR. The MSE and R^2^ of the SSC retrieval model of VBR amplitude were 2.28 mg/L and 0.906, respectively. The distribution of the SSC of the entire research area could be retrieved by inputting the VBR amplitudes extracted via waveform decomposition (Figure 9b) into the SSC retrieval model (Equation (12)). Compared with exponential regression and ANN-based SSC models of ALB depth bias, the waveform decomposition method showed a higher SSC retrieval accuracy. The shortcomings of the waveform decomposition method are that it comprises complex waveform processing procedures and the laser waveforms are not always available for users.
  In summary, the MSEs of the exponential SSC regression model of depth bias, the power SSC regression model of VBR amplitude, and the proposed ANN-based SSC model of depth bias were 15.28, 2.28, and 2.564 mg/L, respectively. The waveform decomposition method presented the highest SSC retrieval accuracy among the three SSC models. The accuracy of the ANN-based SSC model of depth bias was higher than that of the exponential regression SSC model of depth bias because the neural network was able to build more precise connections between ALB depth bias and SSC than the traditional regression method.

### 4.2. Advantages and Limitations

The advantages of the proposed method are as follows: (1) Compared with the waveform decomposition method, the proposed SSC retrieval method using ALB depth bias does not require a complex waveform processing procedure and is easy to conduct. (2) Compared with the exponential SSC regression model of depth bias, the proposed ANN-based SSC model of depth bias has a higher SSC retrieval accuracy. (3) Compared with the measurement bias method using NWSP, the proposed SSC retrieval method using depth bias does not require the help of infrared lasers and can be generally suitable for single- and dual-wavelength ALB systems.

The limits of the proposed method are summarized as follows: (1) Since the ALB capacity is significantly affected by turbidity, the question of whether the ALB bottom point height *h*_ALB_ can be obtained is essential for the calculation of depth bias Δ*h*. With the exception of extremely turbid water, ALB can realize water bottom detection and provide *h*_ALB_. (2) Single-beam echo sounding cannot realize full-coverage measurements but can only provide information on discrete points. ALB depth bias values calculated by taking single-beam echo sounding data as a reference can be obtained at those discrete points. Therefore, SSC inversion using depth bias cannot realize planar inversion but only discrete-point inversion, as shown in Figure 7b. This limitation can be overcome by taking multibeam sonar data as a reference in the future.

### 4.3. Generalization Ability

ANN-based modeling has a certain randomness, such as the random division of the training, validation, and testing data. One trained model may not reflect the real performance because of randomness. To evaluate the generalization ability of the SSC model, multiple models should be trained. The MSE and *R* of each model can be calculated, and the mean values of MSE and *R* can be used to assess the generalization ability of the ANN-based SSC model. We obtained five models using the same datasets and parameter settings, and the MSE and *R* of each model of the testing data are shown in Table 3. The mean values of MSE and *R* were 2.194 mg/L and 0.966, respectively. The results show that the ANN-based SSC model had a good generalization ability.

## 5. Conclusions and Suggestions

In this study, we proposed a novel method for SSC retrieval using the depth bias of airborne bathymetric LiDAR data. The depth bias of ALB was used as an indicator of SSC, and an ANN was used to build an empirical SSC model by connecting the ALB depth bias and the measured SSC. The proposed method was verified using a dataset collected from the Optech CZMIL system. The results verified the effectiveness of SSC prediction using ALB depth bias.

Compared with the waveform decomposition method, this method does not require waveform data, which are not always available for users. Compared with the exponential SSC model of depth bias, the proposed ANN-based SSC model of depth bias has a higher accuracy. Compared with the measurement bias method using the NWSP, this method does not require the help of an infrared laser. The proposed method provides a new method for SSC inversion using ALB when waveform data or infrared laser data are not available. The results also reveal that ALB can provide additional environmental information when used for shallow water measurements.

SSC inversion using depth bias, calculated by taking single-beam echo sounding data as a reference, can be performed to obtain SSCs at discrete points. Multibeam sounding data should be used to calculate ALB depth bias to realize full-coverage SSC inversion. The ANN architecture used in the present study, containing one hidden layer, is simple and easy to use. Future studies should be conducted to explore different ANN architectures to further improve the accuracy of SSC retrieval using ALB depth bias.

## Figures and Tables

**Table 1:** Statistical parameters of raw data used for model construction.

Parameter	Min	Max	Mean	Std
Depth bias (m)	−0.305	1.221	0.161	0.28
Depth (m)	3.022	4.559	3.366	0.314
Scanning angle (degree)	15.553	20.938	19.052	1.021
Sensor height (m)	394.007	441.333	420.381	10.701
SSC (mg/L)	164.087	193.044	176.591	5.587

**Table 2:** Datasets and performance of the ANN-based SSC model.

Datasets	Samples	MSE (mg/L)	*R*
Training	218	0.421	0.993
Validation	72	1.248	0.985
Testing	72	2.564	0.960

**Table 3:** Generalization ability of the SSC model.

Model Number	MSE (mg/L)	*R*
1	2.564	0.960
2	2.026	0.976
3	2.548	0.954
4	1.750	0.971
5	2.080	0.969

## Data Availability

The datasets generated and/or analyzed during the current study are available from the corresponding author on reasonable request.
